# Augmented reality guided osteotomy in hallux Valgus correction

**DOI:** 10.1186/s12891-020-03373-4

**Published:** 2020-06-17

**Authors:** Arnd Fredrik Viehöfer, Stephan Hermann Wirth, Stefan Michael Zimmermann, Laurenz Jaberg, Cyrill Dennler, Philipp Fürnstahl, Mazda Farshad

**Affiliations:** 1 Department of Orthopaedics, Balgrist University Hospital, Forchstrasse 340, 8008 Zürich, Switzerland; 2 Computer-Assisted Research and Development Group, Balgrist University Hospital, Zurich, Switzerland

**Keywords:** Augmented reality, First ray shortening, Transfermetatarsalgia, Hallux valgus

## Abstract

**Background:**

An optimal osteotomy angle avoids shortening of the first metatarsal bone after hallux valgus surgery and therefore reduces the risk of transfer-metatarsalgia. The purpose of the present ex-vivo study was to investigate whether augmented reality (AR) would improve accuracy of the distal osteotomy during hallux valgus surgery.

**Methods:**

Distal osteotomies of the first metatarsals were performed on a foot model by two surgeons with different levels of surgical experience each with (AR, *n* = 15 × 2) or without (controls, *n* = 15 × 2) overlay of a hologram depicting an angle of osteotomy perpendicular to the second metatarsal. Subsequently, the deviation of the osteotomy angle in the transverse plane was analyzed.

**Results:**

Overall, AR decreased the extent of deviation and the AR guided osteotomies were more accurate (4.9 ± 4.2°) compared to the freehand cuts (6.7 ± 6.1°) by tendency (*p* = 0.2). However, while the inexperienced surgeon performed more accurate osteotomies with AR with a mean angle of 6.4 ± 3.5° compared to freehand 10.5 ± 5.5° (*p* = 0.02), no significant difference was noticed for the experienced surgeon with an osteotomy angle of around 3° in both cases.

**Conclusion:**

This pilot-study suggests that AR guided osteotomies can potentially improve accuracy during hallux valgus correction, particularly for less experienced surgeons.

## Background

Hallux valgus deformity is a one of the most common deformities of the foot [1]. Once conservative measures have failed, distal first metatarsal (MT I) osteotomies (e.g. ReveL) are well established techniques to correct hallux valgus deformities [2–6]. The principal technique of these operations is to osteotomize the MT I and to shift the distal part laterally along the plumb line of the second metatarsal in the transverse plane [4]. Accurate performance of the osteotomy is mandatory to avoid additional shortening of the MT I. Geometrical analysis has shown that a posterior deviation of only 10° shortens the MT I by 5 mm and might therefore lead to transfer metatarsalgia postoperatively. Various technologies have been employed to increase the accuracy in implementation of the optimal planning, but their clinical application has been challenged by increased surgical time, exposure, as well as need for additional costly infrastructure or slow learning curves. A recent promising technological advancement is augmented reality (AR), making a superimposition of a hologram to reality possible, providing additional information to the performing surgeon. This can be realized by a semitransparent mirror which allows the surgeon to see the generated images projected onto real objects in so called “Video See-Through Head-Mounted Display “(VSTHMD) systems. The first AR VSTHMD was built in 1968 by Sutherland [7]. More recent improvements in AR allow to display generated images in real time on small head mounted displays (e.g. Hololens™), making AR now feasible for use in the operation theatre. AR has been evaluated in several surgical fields [8–13], but to our knowledge, no study has yet evaluated the use of AR in foot surgery.

The purpose of the present ex-vivo study was to investigate whether overlaying a hologram to reality would improve accuracy of the distal osteotomy for hallux valgus surgery.

We hypothesized that overlaying a hologram will increase the accuracy of distal MT I osteotomy, especially when carried out by inexperienced surgeons.

## Methods

### The foot models

**Fig. 1:**
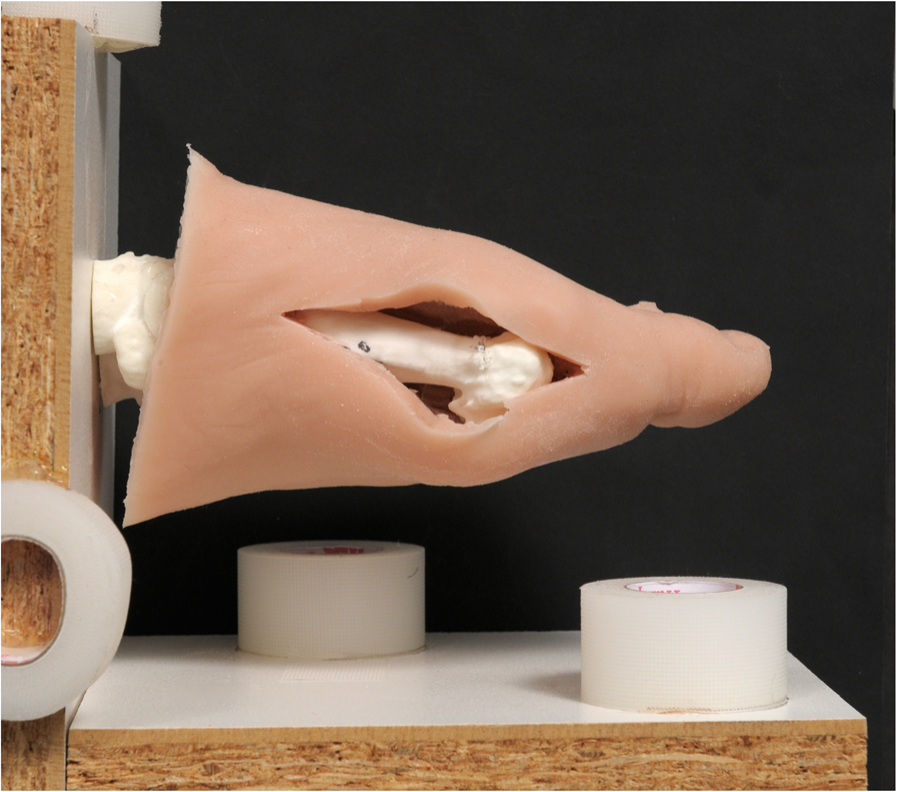
Foot dummy. The approach through the silicon reveals shows the exchangeable polyamide MT I

The experiments were performed on a foot dummy consisting of a polyamide forefoot skeleton with an exchangable first metatarsal bone (MT I) with a soft tissue cover, made of medical silicone (Fig. 1).

To build the 3 dimensional forefoot skeleton, a CT scan (Philips Brilliance 40 CT, Philips Healthcare, The Netherlands) from a cadaveric foot with hallux valgus deformity was conducted under axial load of 350 N in a custom made clamping system. The data of the 3 dimensional model of the foot were segmented and implemented in the preoperative planning software CASPA (version 4.18, Balgrist CARD AG, Zurich, Switzerland). The foot showed a moderate hallux valgus deformity with an intermetatarsal angle (IMA) of 15°. In order to obtain three different hallux valgus manifestations (slight, moderate and severe), the MT I was rotated parallel to the plantar plate to an IMA of 18° (severe hallux valgus) and 13° (slight hallux valgus) through the center of the sphere defined by the proximal articular surface of the MT I. A pluggable connector was incorporated in the model between the MT I and the medial cuneiform bone to permit exchange of the MT I after completion of an osteotomy. The polyamide skeleton was printed with 60 replaceable MT I (with 20 slight, 20 moderate and 20 severe hallux valgus manifestations) using selective laser sintering [14].

The soft tissue cover of the foot dummy model was generated by using a plaster cast mold of the forefoot and midfoot of the cadaver. The cast was lined with a dividing layer (Body Double, SmoothOn, Macungie PA, USA) and filled with medical silicon (Elastosil, Wacker, Riemerling, Germany). The polyamide skeleton model was subsequently immerged in the silicone before the silicon had cured.

A dorsomedial approach through the silicone cover to the MT I was performed (AFV and SMZ). Finally, the model was fixed to a wooden frame with 3 dimensional markers attached to it for facilitated alignment of the hologram.

**Fig. 2:**
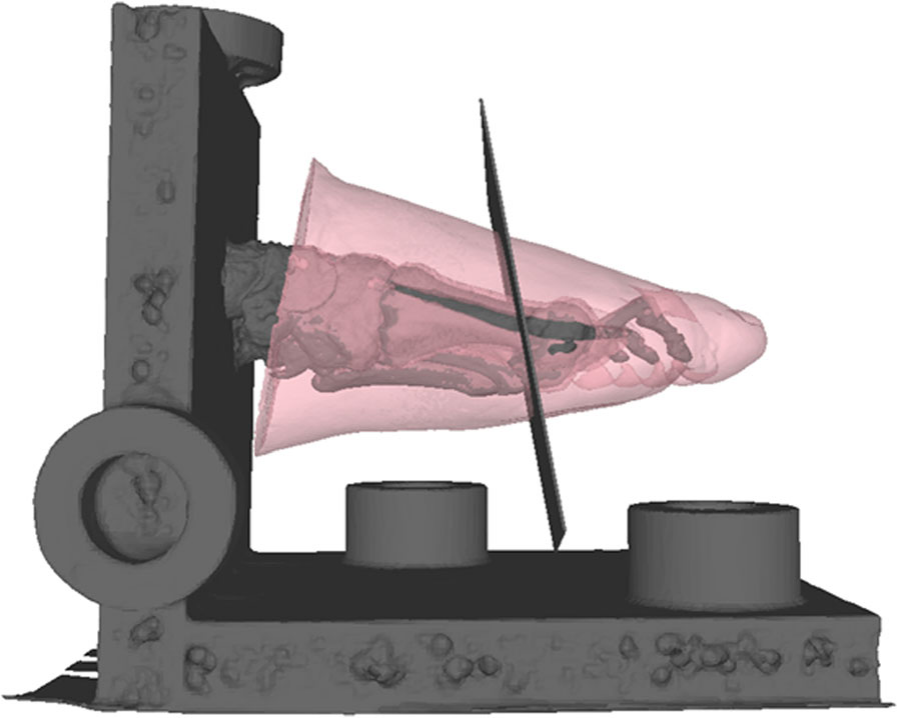
3 dimensional model of the foot: The hologram model projected in the experiments. The plane guides an osteotomy perpendicular to the second metatarsal

For the augmented reality (AR) experiment, a cutting plane perpendicular to the axis of the second metatarsal bone and perpendicular to the plantar plate was integrated in the model to serve as cutting guide in the experiments (Fig. 2). The model for slight, moderate, and severe hallux valgus, including the cutting plane and the model of the frame and its markers, were then transferred to the head mounted device (HMD) (Hololens™, Microsoft Corporation, Redmond, WA 98052–6399, USA)).

### Experiments

**Fig. 3:**
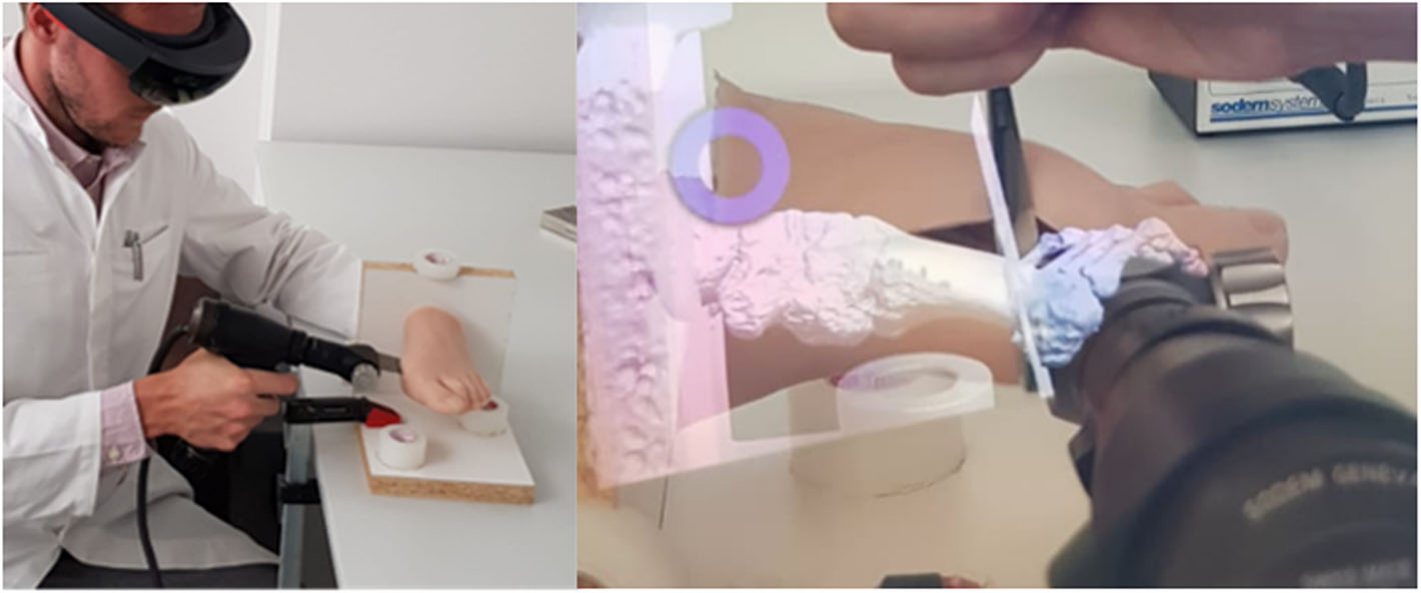
Setup of the experiments with augmented reality. Left: Surgeon performing the osteotomy wearing the Hololens™^.^ Right: foot model with overlaying hologram

The distal osteotomy of the MT I was performed using an oscillating saw blade (Sodem Systems, Geneva, Switzerland). The cuts were alternately carried out freehand or with an overlaid hologram wearing the HMD (Fig. 3). They were performed by an experienced surgeon in foot and ankle surgery and an unexperienced surgeon. The experienced surgeon (SMZ) had worked as a consultant in the foot and ankle department for 2 years and performed hallux valgus surgery on a daily routine. The inexperienced surgeon was a resident (LJ) who had no prior experience in hallux valgus surgery at the time of the experiments. The experiments were randomized concerning freehand or AR cuts and the manifestation of the hallux valgus deformity. Each surgeon performed 15 osteotomies with HololensTM and 15 freehand osteotomies with randomized degree of deformity. In an attempt to avoid adaptation to the setting, a maximum of 6 experiments was set for each surgeon per day.

### Analysis

**Fig. 4:**
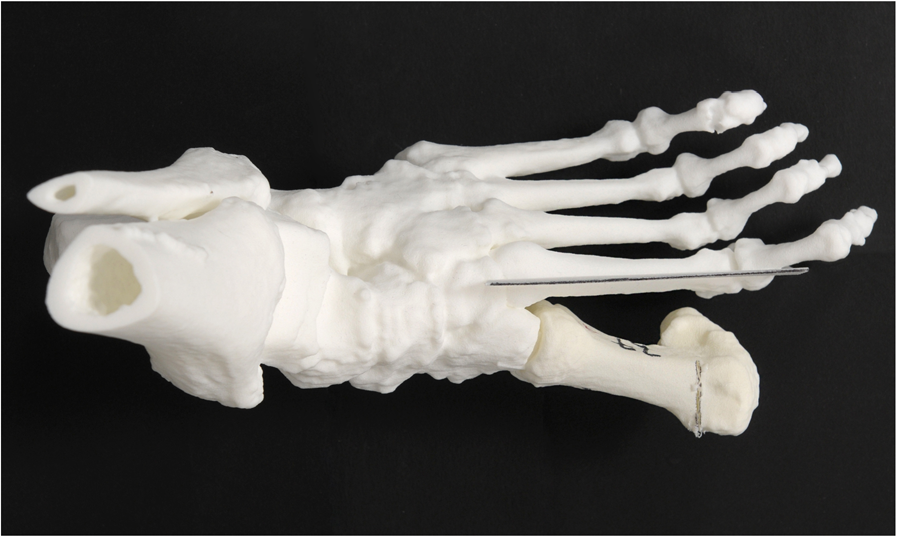
Skeletal model for analysing the osteotomie angle in relation to axis of the second metatarsal (black line). An osteotomised first metatarsal is shown exemplary

A second skeletal model (Fig. 4) was printed for analysis without the first metatarsal but a plane perpendicular to the plantar plane and parallel to the axis of the second metatarsal. The osteotomized first metatarsals of the experiments could be connected to the analysis skeleton model through a plug. A picture of each MT I osteotomy was taken perpendicular to the plantar plate and centered over the distal first metatarsal with a camera (Nikon, Tokyo, Japan). The angle between the axis of the second metatarsal and the osteotomy was measured using the measurement tool in Synedra (Synedra Schweiz AG, Dübendorf, Switzerland). The deviation in other planes than the plantar plane were not considered as they don’t account for changes in MT I length postoperatively. The measurements were carried out by two independent observers blinded to the surgical method and surgeon that performed the osteotomy (LJ and AFV). The statistical analysis was performed using SPSS (IBM, Armonk, NY, USA). The Student’s T test for independent samples was used to determine whether the differences were significant (*p* < 0.05), assuming normal distribution of data. Cronbach’s Alpha was used to measure internal consistency (inter observer correlation). A post hoc power analysis was performed with G*Power (Heinrich-Heine-University, Düsseldorf, Germany).

### Source of funding

ResOrtho Foundation, University Hospital Balgrist. Funding source did not play a role in this investigation.

## Results

**Fig. 5:**
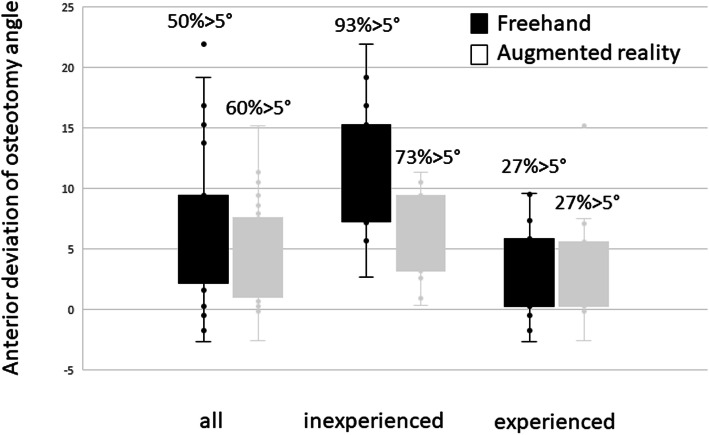
Deviation of osteotomy angle. Outliers > 5° are given in percentage for each setup. Left: Osteotomy angle for both surgeons. The results for AR (mean angle 4.1°) and freehand osteotomies (mean angle 6.1 °) did not differ significantly (*p* = 0.2), but the variability of imprecision is less with AR-support. Middle: Osteotomy angle for the experienced surgeon. The results for the AR (mean angle 3.4°) and freehand (mean angle 2.8°) osteotomies did not differ significantly (*p* = 0.2). right: Osteotomy angle for the inexperienced surgeon. The osteotomy angle for the AR (mean angle 6.4°) differed significantly (*p* = 0.02) less from the plane perpendicular to the second metatarsal than the freehand osteotomies (mean angle 10.5°). Also the variability of imprecision is higher without AR-support

Overall, the mean deviation between the osteotomy plane and the target plane perpendicular to the second metatarsal was 4.9 ± 4.2° in anterior direction (i.e. from proximal medial to distal lateral) with help of AR, and 6.7 ± 6.1° in anterior direction for freehand osteotomies. The difference between the osteotomy angle between freehand performance and experiments with AR was not significant if the combined average performances of both surgeons were considered (*p* = 0.2) (Fig. 5). However, AR decreased the extent of deviation with a smaller standard deviation (regular 6.1°, AR 4.2°) and a smaller range (regular 25°, AR 18°).For the more experienced foot surgeon, the use of AR did not increase the accuracy of distal osteotomies significantly overall. The mean deviation from the plumb line of the osteotomy to the second metatarsal was 3.4 ± 4.3° (range 18) with AR and 2.8 ± 3.8° for the freehand osteotomies (range 11, (Fig. 5). For the less experienced surgeon, the osteotomies with AR were significantly (*p* = 0.02) more accurate with an angle of 6.4 ± 3.5° (range 19) compared to the freehand osteotomies with an angle of 10.5 ± 5.5° (range 12, Fig. 5). The maximum deviation for the less experienced surgeon was 22° for the freehand and 11° for the AR supported osteotomies.

**Fig. 6:**
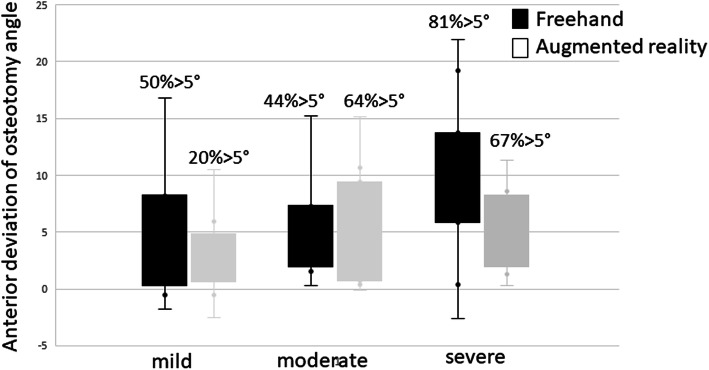
Results for freehand and Hololens™ osteotomies for different hallux valgus manifestations. Outliers > 5° are given in percentage for each setup. Left: mild hallux valgus. Middle: moderate hallux valgus. Right: severe hallux valgus

Although the more experienced surgeon also achieved better results with AR-support than the less experienced surgeon, this finding was not significant enough in our series of experiments (*P* = 0.051). There was no significant difference in the precision of osteotomy angles for the different simulated manifestation of the deformity Fig. 6.

The reported mean deviations imply an anterior deviation in regard to the plumb line of the second metatarsal bone. A posterior deviation was only observed in 10% (6/60. AR 3, regular 3) of our cases.

The analysis showed excellent inter-observer reliability with a Cronbach’s Alpha of 0.978.

The post hoc power analysis stated a power 1-β of 0.92 for an effect size d of 0.8 and a power of 0.61 for an effect size of d = 0.5 (α = 0.05).

## Discussion

The aim of the present study was to investigate whether AR improves accuracy of the osteotomy angle in distal hallux valgus surgery. We hypothesized that overlaying a hologram helps surgeons perform the osteotomy more accurately, especially when inexperienced. We present the first report on potential merits of a promising new technology (AR) in a randomized ex-vivo study, and report the accuracy of the distal osteotomy during hallux valgus surgery with and without the support of this new technology with different levels of surgical experience.

When viewing the results for the two surgeons individually, the experienced surgeon did not perform the osteotomies more accurately with help of AR. The inexperienced surgeon however obtained better results when using AR technology. The accuracy of the osteotomy angle improved significantly for the osteotomies with AR (*p* = 0.02) by 4°. Without AR, the osteotomy revealed a mean deviation of 10.5° from the plane perpendicular to the second metatarsal. In accordance, the range of accuracy was much larger without AR-support. This was also true if the results of both surgeons were considered. The here observed important differences outline the power of AR guided osteotomies to decrease the learning curve of less experienced surgeons. This finding is in line with the results of other types of navigated surgery [15]. A correct osteotomy angle can avoid shortening of the first metatarsal bone after hallux valgus surgery and therefore reduce the risk of transfer-metatarsalgia. According to geometrical considerations, a posterior deviation of 10° to the plane perpendicular to the second metatarsal may shorten the MT I by up to 5 mm for correction of severe hallux valgus. With the current technique of free-hand osteotomies, an unwanted variability in accuracy is observed. The improved osteotomy angle with AR-support for the inexperienced surgeon would reduce shortening of the MT I by 20% to around 4 mm if the divergence would be in the posterior direction. This would even be more pronounced if the differences between the expected shortening for the maximum deviation of the osteotomy angle (22° freehand and 11° AR) are analyzed: For the inexperienced surgeon, the difference would be 3 mm or 38% (8 mm freehand and 5 mm AR of MT I shortening, respectively). Although the correlation of amount of shortening of the MT I to transfer-metatarsalgia might not be direct, recent studies have shown an increased risk for metatarsalgia in patients with shortening of as little as 2 mm [16]. For an anterior deviation of the osteotomy angle, as observed in the present study, the differences in length become distinctly smaller. Of notice, we routinely record a tendency toward posterior deviation for freehand cuts in our clinical observation in contrast to the anterior deviation observed in the present study. This could be explained due to the model set up, which is the main limitation of this ex vivo experiment.

The power analysis showed a sufficient power for an effect size of 0.8, but not for an effect size of 0.5. The observed tendency towards a higher accuracy for AR performed osteotomies for both surgeons might become statistically significant for higher sample sizes. We believe an effect size of 0.8 to be sufficient, as small deviations in the osteotomy angle are not clinically significant.

Furthermore, a higher sample size in this experimental set up also risks that the freehand osteotomies are performed more accurately due to a learning curve resulting from the AR guided osteotomies.

Although a lot of effort was made to create a realistic foot model, artificial bones with a soft tissue cover were used. It is likely that the alignment of the hologram, although simplified by the frame of the foot, is a source of error. Further sources of error include precision of the augmented reality equipment, and performing the osteotomy guided by the hologram. Finally, the present study cannot quantitively assess the influence of each source of error Advancements of the holographic technology already allow a better alignment of the hologram.

## Conclusion

Considering the experimental setup, we conclude that AR is a powerful technology with the potential to improve accuracy in hallux valgus correction, particularly for less experienced surgeons. Following the here presented first report of use of AR in foot surgery, clinical studies are necessary to verify whether this promising technology will improve the outcome following hallux valgus surgery.

## Data Availability

The datasets used and/or analysed during the current study are available from the corresponding author on reasonable request.

## Abbreviations

AR: Augmented reality
HMD: Head mounted device
IMA: Intermetatarsal angle
MT I: First metatarsal bone
VSTHMD: Video see-through head-mounted display
